# Osimertinib-Induced Myositis in a Patient With Metastatic Non-small Cell Lung Cancer With Epidermal Growth Factor Receptor Mutation

**DOI:** 10.7759/cureus.67597

**Published:** 2024-08-23

**Authors:** Aatma Ram, Chun T Siu, Abir Mukherjee, Ranju Gupta

**Affiliations:** 1 Hospice and Palliative Medicine, Lehigh Valley Health Network, Allentown, USA; 2 Internal Medicine, Rosalind Franklin University of Medicine and Science, McHenry, USA; 3 Hematology and Medical Oncology, Lehigh Valley Health Network, Allentown, USA; 4 Pathology, Lehigh Valley Health Network, Allentown, USA

**Keywords:** rhabdomyolysis, tki, myositis, osimertinib, osimertinib induced myositis

## Abstract

Osimertinib is a third-generation tyrosine kinase inhibitor (TKI) that has emerged as a standard treatment in non-small cell lung cancer (NSCLC) with epidermal growth factor receptor (EGFR) mutation. While it is generally well tolerated, milder side effects of diarrhea, cytopenia, and cutaneous rashes are common. Osimertinib-induced myositis and rhabdomyolysis are exceedingly rare, and only a few cases have been documented in the literature to date. In this report, we present a case of a 59-year-old female with metastatic NSCLC who experienced myalgia following the initiation of osimertinib. Blood work revealed elevated creatine kinase (CK), serum creatinine (Cr), alanine aminotransferase (ALT), and aspartate aminotransferase (AST). Initially, her myalgia improved, and lab work normalized after drug discontinuation and supportive care. However, rechallenge with a 50% dose resulted in recurrence of symptoms and elevated serum CK, Cr, ALT, and AST. MRI findings suggested diffuse inflammation and a muscle biopsy revealed necrotizing myopathy. Symptoms ameliorated upon complete cessation of the drug and use of steroids. This case highlights the importance of recognizing this rare adverse effect of osimertinib and a guide for managing these associated symptoms.

## Introduction

Lung cancer is the most common cause of cancer-related deaths globally, affecting both men and women [1]. Among lung cancers, non-small cell lung cancer (NSCLC) constitutes 84% of cases with major subtypes, including adenocarcinoma, squamous cell carcinoma, and large cell carcinoma [2]. Treatment modalities for these cancers vary but commonly include surgery, chemotherapy, and radiotherapy. Significant advancements in molecular translational research over the past two decades have significantly enhanced our understanding of cancer biology. The development of tyrosine kinase inhibitors (TKIs) to target gene mutations has revolutionized cancer treatment [3-5]. Notably, one such genetic alteration involves activating mutation in the epidermal growth factor receptor (EGFR) gene. EGFR plays a crucial role in various signaling pathways associated with cell growth, differentiation, and survival. Osimertinib is an EGFR TKI that has emerged as a well-tolerated therapeutic option for NSCLC characterized by the EGFR Exon 21 L858R mutation. While generally well tolerated, uncommon side effects like myositis and rhabdomyolysis are increasingly reported with the rising use of this medication [6-11].

## Case presentation

A 59-year-old woman developed a radiographic progression of the disease in April 2023. A PET-CT scan followed an abnormal surveillance CT chest, revealing bilateral pulmonary nodules and new osseous involvement in the bilateral iliac bones. Her medical history included stage IIIa NSCLC diagnosed in 2019, for which she underwent right partial lung resection and mediastinal lymph node dissection, followed by adjuvant radiation therapy. Additionally, she had a history of chronic pain syndrome, fibromyalgia, and reflex sympathetic dystrophy from a previous work-related injury. These symptoms were managed with stable doses of chronic opioid therapy, including extended-release (ER) morphine and oxycodone immediate release (IR), under pain management care. 

A CT needle biopsy of a new lung nodule confirmed metastatic lung primary adenocarcinoma. Despite a known EGFR Exon 21 L858R mutation, she had previously opted against EGFR-targeted therapy and preferred a watchful approach over the newly FDA-approved osimertinib in 2020. However, with the finding of metastatic recurrence, she was started on osimertinib. Initial treatment was well tolerated, but one month later, she developed myalgia prompting osimertinib cessation. Due to worsening myalgia and muscle cramping, she presented to the emergency room (ER) for further evaluation. Laboratory findings are detailed in Table 1. 

**Table 1:** Blood work. Lab work was drawn in the ER upon the first ER visit. ER: emergency room.

Lab	Value	Reference values
Creatine kinase (CK)	14729 U/L	30-223 U/L
Creatinine (Cr)	1.83 mg/dL	0.4-1.10 mg/dL
Alanine transaminases (ALT)	655 U/L	<41 U/L
Aspartate transaminases (AST)	827 U/L	<41 U/L

The abnormal lab values of serum CK, Cr, ALT, and AST were concerning for rhabdomyolysis. Consequently, the patient was hospitalized for IV hydration and close lab monitoring. Subsequent laboratory tests showed normalization of all lab work.

Considering similar case reports noted in the literature and after discussion with the patient, a decision was made to rechallenge osimertinib at a 50% dose reduction while monitoring serum CK levels weekly. Myalgias recurred two weeks after restarting osimertinib, leading to its immediate discontinuation. Myalgia worsened despite discontinuing osimertinib and outpatient IV hydration, prompting an ER visit. The patient was hospitalized for a workup, and a rheumatology consult was obtained. Blood work is detailed in Table 2. 

**Table 2:** Blood work. Lab work was drawn in the ER upon the second ER visit. ER: emergency room, SRP: signal recognition particle.

Lab	Value	Reference values
Creatine kinase (CK)	23600 U/L	30-223 U/L
Creatinine (Cr)	1.48 mg/dL	0.4-1.10 mg/dL
Alanine transaminases (ALT)	598 U/L	<41 U/L
Aspartate transaminases (AST)	250 U/L	<41 U/L
Erythrocyte sedimentation rate (ESR)	30 mm/hr	0-30 mm/hr
C-reactive protein (CRP)	5.5 mg/dL	<10 mg/dL
C3 complement	136 mg/dL	87-200 mg/dL
C4 complement	46 mg/dL	19-52 mg/dL
Antinuclear Abs (ANA)	Absent	Absent
Hepatitis B surface antigen	Nonreactive	Nonreactive
Hepatitis C antibody profile	Nonreactive	Nonreactive
3-Hydroxy-3-methylglutaryl coenzyme A reductase	<3	0-19
Myositis specific panel	-	-
Jo-1 autoantibody	0	0-40 AU/mL
P155/140 Ab	Negative	Negative
EJ antibody	Negative	Negative
Mi-2 Ab	Negative	Negative
OJ Ab	Negative	Negative
PL-7 Ab	Negative	Negative
PL-12 Ab	Negative	Negative
SRP Ab	See note	Negative
The immunoprecipitation assay shows a band migrating around 54 kDa. Therefore, the presence of SRP antibodies cannot be ruled out.		

**Figure 1:**
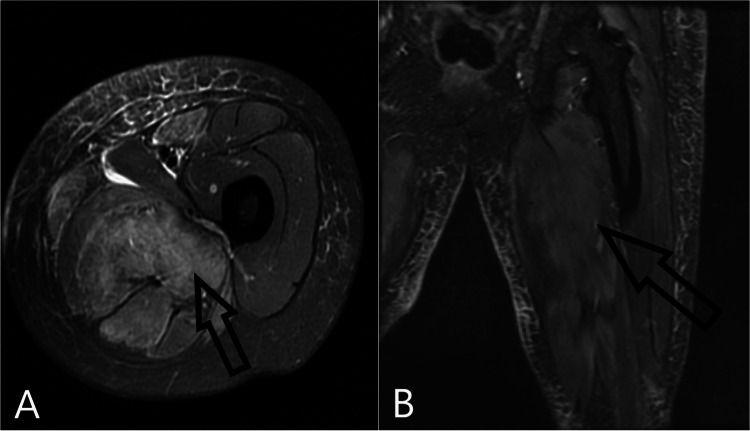
MRI of the left thigh. (A) Axial plane and (B) Coronal plane. Arrows highlight diffuse edema in left thigh muscles in different MRI planes.

MRI of the left thigh was obtained and revealed diffuse intramuscular edema throughout the left thigh muscles, favoring diffuse myositis. See the images in Figure 1.

**Figure 2:**
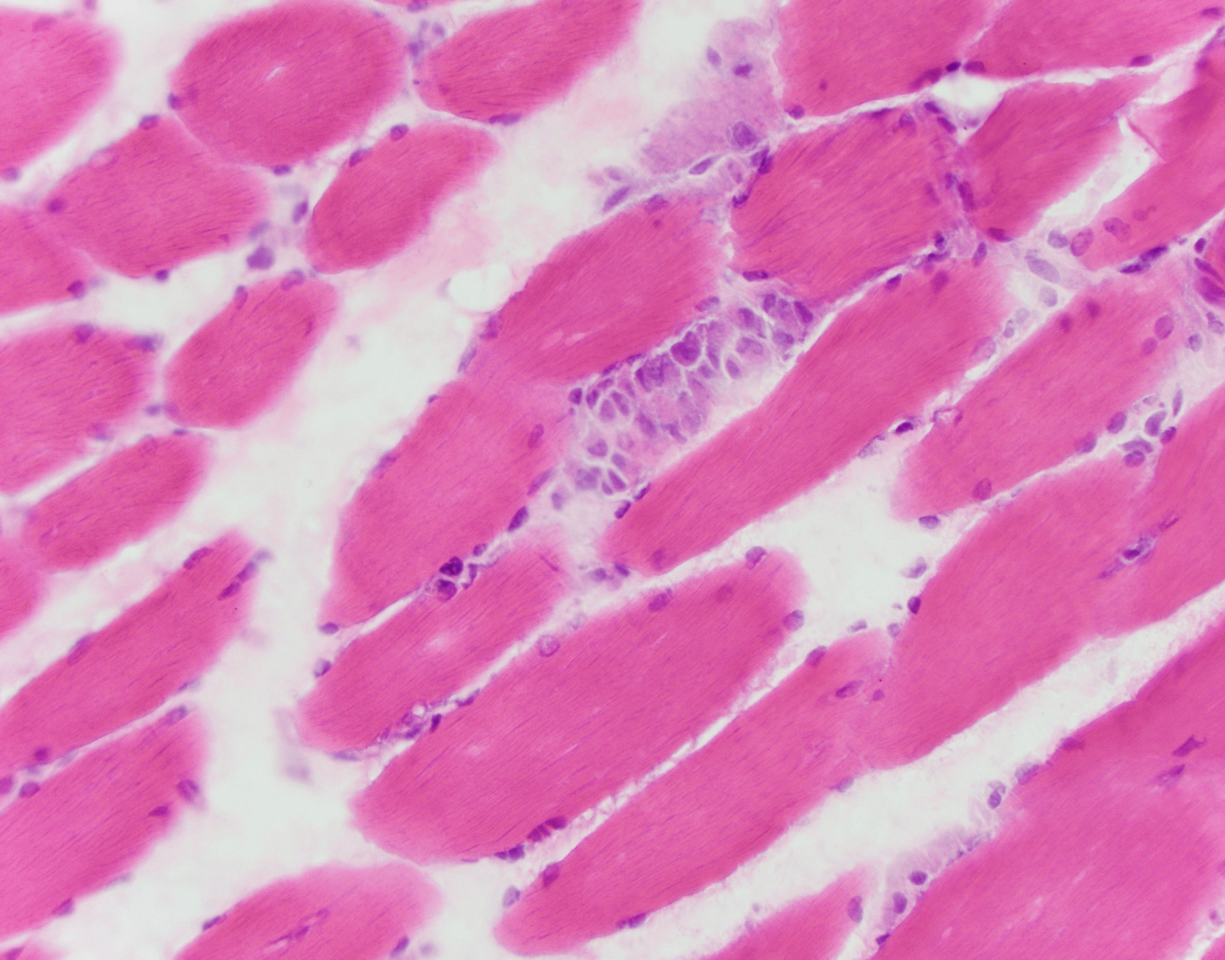
Muscle biopsy of thigh.

Based on these findings, a muscle biopsy was performed, and empiric steroids with prednisone 40 mg daily were started to treat inflammatory myositis. Figure 2 shows the thigh muscle biopsy, which revealed necrotizing myopathy with myophagocytosis and upregulation of MHC-1.

**Figure 3:**
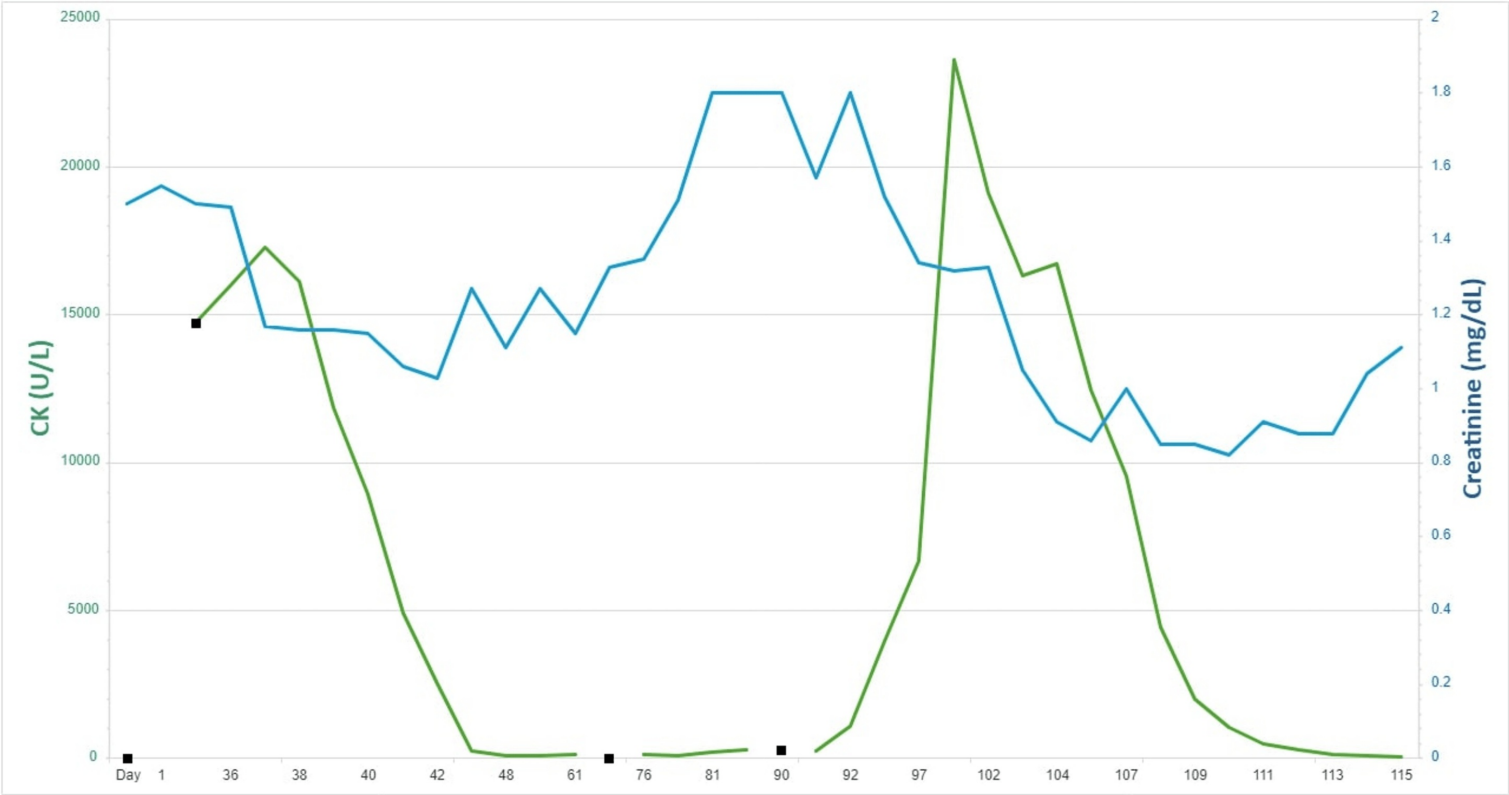
Serum CK and Cr levels over time. CK: creatine kinase, Cr: serum creatinine.

She remained hospitalized for over two weeks while receiving supportive care and steroids. Her myalgia slowly subsided, and lab work returned to normal. Figure 3 details the fluctuations in serum CK and Cr levels in correlation with osimertinib use.

In a follow-up visit with rheumatology a month after hospitalization, the patient tapered off prednisone, and a repeat myositis-specific 12 antibody panel was negative, including an anti-SRP antibody. The patient continues to follow up in our oncology clinic and started on Erlotinib 100 mg daily for more than three months now with good tolerance and no symptoms of myositis. 

## Discussion

Osimertinib, an irreversible tyrosine kinase receptor inhibitor, is the standard treatment for previously untreated advanced NSCLC and as adjuvant therapy for patients with stage IB to IIIA NSCLC with EGFR Exon 19 deletions or EGFR Exon 21 L858R mutations, based on ADURA and FLAURA trials [12,13]. Additionally, the LAURA trial showed a progression-free survival benefit with osimertinib in unresectable stage III NSCLC after chemoradiotherapy [14]. Although our patient had EGFR L858R mutation, she did not receive adjuvant osimertinib for her Stage IIIA NSCLC at diagnosis in 2019 because the results of the ADURA trial were published in October 2020, and FDA approval came in December 2020. Subsequently, upon approval of osimertinib, the patient opted for a watchful approach. However, upon later confirmation of metastatic recurrence through biopsy, the patient started osimertinib. 

Osimertinib is rarely associated with serious adverse events, and commonly reported adverse events during trials included diarrhea (58-60%), cutaneous rashes (58-59%), nail changes (35-39%), and stomatitis (29%) [8,9]. Myalgia was reported in 10% of patients, while 7-10% of patients developed mildly elevated liver enzymes, all without life-threatening myositis or musculoskeletal complications [8,9]. In contrast, our patient experienced severe myalgia and necrotizing myopathy accompanied by elevated hepatic transaminases and serum creatinine. 

The interval between osimertinib initiation and myalgia occurrence is variable, occurring between four days and eleven months after drug initiation [6,7]. Our patient developed myalgia four weeks after starting the drug, leading to its cessation and supportive care. A rechallenge at 50% dose caused a recurrence of symptoms in only two weeks, necessitating drug discontinuation.

While prevalence studies are scarce, osimertinib-induced myositis is documented in individual case reports and small case series. Parafianowicz et al. reported that four out of thirty-eight NSCLC patients treated with osimertinib developed myositis. Two of these patients required discontinuation of the drug, while the other two patients continued osimertinib under close monitoring [7]. Fujioka et al. highlighted a case that showed improvement following dose adjustment [8]. Conversely, Crowley et al. reported a case of life-threatening myositis shortly after initiation of osimertinib, where symptoms persisted despite drug discontinuation and treatment with steroids and IVIG [6]. Although our patient developed myositis, a favorable response ensued after drug discontinuation. Unlike some previously reported cases, our patient did not tolerate a rechallenge with a reduced medication dose. Despite the empiric use, the role of steroids in our case remains questionable for two reasons: first, the patient had previously shown resolution of myositis and correction of lab abnormalities without steroid use; second, most inflammatory markers were negative, which raised doubts about other immune-mediated etiologies.

The exact pathophysiology of osimertinib-induced myositis remains unknown. However, leading hypotheses include immune-mediated muscle damage and drug-induced inhibition of tyrosine kinase receptors in myocytes, which may lead to cell senescence and apoptosis [13,15]. We also suspect a potential role of drug-drug interactions, as our patient was concurrently taking oxycodone, which is metabolized via the CYP34A system, the same pathway involved in processing osimertinib [16]. Additionally, while statins are commonly implicated in drug-induced myopathies, our patient was not using any statins. Nonetheless, no dose adjustment for osimertinib has been recommended to date for patients concurrently taking oxycodone. Despite the theoretical interaction between osimertinib and oxycodone, further research is warranted to elucidate the etiology of osimertinib-induced myositis and the role of drug-drug interaction. It should be noted that our patient was switched over to another EGFR tyrosine kinase inhibitor, Erlotinib, with good tolerance suggesting no cross-reactivity of this adverse event.

## Conclusions

Drug-induced myositis ranks among the most common causes of myositis. Manifestations can vary in severity, ranging from mild myalgia, muscle cramping, and asymptomatic CK elevations to life-threatening myositis, rhabdomyolysis, and end-organ dysfunction. Myositis cases have been documented in association with the use of tyrosine kinase inhibitors, including imatinib, dasatinib, nilotinib, sorafenib, lapatinib, gefitinib, and osimertinib. Our patient developed recurrent myositis with active use of osimertinib. Switching her to Erlotinib has not resulted in the same symptoms, suggesting no cross-reactivity of these drugs in our patient. This case highlights the importance of recognizing osimertinib-induced myositis and developing a prompt management plan for this side effect. 

## References

[1] Siegel RL, Miller KD, Jemal A (2020). Cancer statistics, 2020. CA Cancer J Clin.

[2] Ganti AK, Klein AB, Cotarla I, Seal B, Chou E (2021). Update of incidence, prevalence, survival, and initial treatment in patients with non-small cell lung cancer in the US. JAMA Oncol.

[3] Lynch TJ, Bell DW, Sordella R (2004). Activating mutations in the epidermal growth factor receptor underlying responsiveness of non-small-cell lung cancer to gefitinib. N Engl J Med.

[4] Pao W, Miller V, Zakowski M (2004). EGF receptor gene mutations are common in lung cancers from "never smokers" and are associated with sensitivity of tumors to gefitinib and erlotinib. Proc Natl Acad Sci U S A.

[5] Arteaga CL (2001). The epidermal growth factor receptor: from mutant oncogene in nonhuman cancers to therapeutic target in human neoplasia. J Clin Oncol.

[6] Crowley F, Fitzgerald BG, Bhardwaj AS, Siraj I, Smith C (2022). Life-threatening myositis in a patient with EGFR-mutated NSCLC on osimertinib: case report. JTO Clin Res Rep.

[7] Parafianowicz P, Krishan R, Beutler BD, Islam RX, Singh T (2020). Myositis-a common but underreported adverse effect of osimertinib: case series and review of the literature. Cancer Treat Res Commun.

[8] Fujioka S, Kitajima T, Itotani R (2018). Myositis in a patient with advanced lung cancer treated with osimertinib. J Thorac Oncol.

[9] Sugimoto H, Matsumoto S, Tsuji Y, Sugimoto K (2022). Elevated serum creatine kinase levels due to osimertinib: a case report and review of the literature. J Oncol Pharm Pract.

[10] Li Y, Liu Y, Zhao Z, Zhang Y (2023). Rhabdomyolysis in a patient with advanced lung cancer treated with osimertinib: a case report. Transl Lung Cancer Res.

[11] Yamasaki M, Matsumoto N, Nakano S (2020). Osimertinib for the treatment of EGFR mutation-positive lung adenocarcinoma complicated with dermatomyositis. Arch Bronconeumol.

[12] Soria JC, Ohe Y, Vansteenkiste J (2018). Osimertinib in untreated EGFR-mutated advanced non-small-cell lung cancer. N Engl J Med.

[13] Wu YL, Tsuboi M, He J (2020). Osimertinib in resected EGFR-mutated non-small-cell lung cancer. N Engl J Med.

[14] Lu S, Kato T, Dong X (2024). Osimertinib after chemoradiotherapy in stage III EGFR-mutated NSCLC. N Engl J Med.

[15] Sakamoto T, Saito Y, Takekuma Y, Kikuchi E, Sugawara M (2023). Gefitinib-induced myositis: a novel case report. Yakugaku Zasshi.

[16] Xu ZY, Li JL (2019). Comparative review of drug-drug interactions with epidermal growth factor receptor tyrosine kinase inhibitors for the treatment of non-small-cell lung cancer. OncoTargets Ther.

